# L-Arginine Intake Effect on Adenine Nucleotide Metabolism in Rat Parenchymal and Reproductive Tissues

**DOI:** 10.1100/2012/208239

**Published:** 2012-04-24

**Authors:** G. Kocic, J. Nikolic, T. Jevtovic-Stoimenov, D. Sokolovic, H. Kocic, T. Cvetkovic, D. Pavlovic, A. Cencic, D. Stojanovic

**Affiliations:** ^1^Institute of Biochemistry, Faculty of Medicine, University of Nis, Bulevar, Dr Zorana Djindjica 81, 18000 Nis, Serbia; ^2^Faculty of Medicine, University of Nis, 18000 Nis, Serbia; ^3^Medical Faculty, University Maribor, 2000 Maribor, Slovenia; ^4^Department of Hygiene, Faculty of Medicine, University of Nis, 18000 Nis, Serbia

## Abstract

L-arginine is conditionally essetcial amino acid, required for normal cell growth, protein synthesis, ammonia detoxification, tissue growth and general performance, proposed in the treatment of men sterility and prevention of male impotence. The aim of the present paper was to estimate the activity of the enzymes of adenine nucleotide metabolism: 5′-nucleotidase (5′-NU), adenosine deaminase (ADA), AMP deaminase, and xanthine oxidase (XO), during dietary intake of L-arginine for a period of four weeks of male Wistar rats. Adenosine concentration in tissues is maintained by the relative activities of the adenosine-producing enzyme, 5′-NU and the adenosine-degrading enzyme-ADA adenosine deaminase. Dietary L-arginine intake directed adenine nucleotide metabolism in liver, kidney, and testis tissue toward the activation of adenosine production, by increased 5′-NU activity and decreased ADA activity. Stimulation of adenosine accumulation could be of importance in mediating arginine antiatherosclerotic, vasoactive, immunomodulatory, and antioxidant effects. Assuming that the XO activity reflects the rate of purine catabolism in the cell, while the activity of AMP deaminase is of importance in ATP regeneration, reduced activity of XO, together with the increased AMP-deaminase activity, may suggest that adenine nucleotides are presumably directed to the ATP regenerating process during dietary L-arginine intake.

## 1. Introduction

 L-arginine is a conditionally essential amino acid important for a number of biochemical functions in protein synthesis, ammonia detoxification, energy fuel via conversion to glucose, the structural component of nucleic acid bound proteins (histones), protein hormones (vasopressin, insulin), polyamines, the component of some enzyme active sites (alkaline phosphatase), component of ejaculate (seminal fluid and sperm), and component of skin and connective tissues proteins (collagen); it is involved in synthesis of creatine and nitric oxide (NO) [1–5]. L-arginine exerts the antioxidant property and the immunomodulatory effect and can reduce the accumulation of ammonia and plasma lactate, toxic byproducts during physical exercise [6–8]. Endogenous L-arginine content is maintained constantly according to the balance between dietary intake, synthesis, and its metabolic pathways. Replenishment of arginine at a similar rate is achieved by a combination of dietary intake and a low rate of endogenous synthesis [9–11].

Adenine nucleotide catabolism and salvage pathway represent important pathways of the intermediary metabolism, as the regulatory effectors (adenosine) or cell energy compound (ATP). The mechanism responsible for the maintenance of optimal adenine nucleotide pool in most tissues interrelates with the AMP metabolism [12]. The initial pathway of AMP metabolism generally occurs via two possible enzyme sequences: the deamination of AMP via enzyme AMP deaminase when IMP is generated or by dephosphorylation via enzyme 5′-nucleotidase (5′-NU) when adenosine is generated [13]. The catabolism of adenosine occurs via adenosine deaminase (ADA) reaction in which inosine is generated. The salvage of performed purines can occur through the utilization of IMP or inosine. Terminal degradation of purine bases is catalyzed via enzyme xanthine oxidase (XO), and the product of catabolism is uric acid. During XO reaction, the free radical species are generated. The regulation of purine metabolism, including the steady-state concentration of adenosine, may be brought about the modifications in the activity of the above-mentioned enzymes [14–16].

 In recent time, the attention is to the use of L-arginine supplementation by athletes, a strategy used widely to enhance tissue growth and general performance, in the treatment of men sterility and prevention of male impotence. Modulation of the arginine-NO pathway through dietary supplementation with L-arginine may be beneficial in the prevention and treatment of the metabolic syndrome in obese humans and in reduction of fat mass [4, 5, 9, 11, 17]. All these functions may be related to the adenylate energy charge, ATP, and adenosine content. The aim of the present study was to estimate the activity of main enzymes of adenine nucleotide metabolism: 5′-NU, ADA, AMP deaminase, and XO during rat dietary intake of L-arginine for a period of four weeks.

## 2. Materials and Methods

### 2.1. Animals

White male Wistar rats (6 months old), 200–220 g body weight, were divided in two groups, where one of them received L-arginine as 0.5% solution dissolved in drinking water, while the other was control. Each group consisted of 8 animals. The rats were sacrificed under Ketalar anesthesia four weeks after. The tissues (liver, kidney, and testis) were quickly removed, rinsed, and homogenized in physiological saline as 1% homogenate. In order to remove cell debris, received homogenate was centrifuged at 600 g on 4°C for 30 min. Received supernatant was used for estimating the enzyme activities and protein concentration.

### 2.2. Enzyme Assays

The activity of 5′-NU was measured according to the method of Wood and Viliams [18] by using 10 mmol/L AMP (Sigma USA) as substrate, where phosphorus liberation was measured. The activity of ADA and AMP deaminase was determined by measuring the ammonia liberation by using 10 mmol/L AMP or 4 mmol/L adenosine (Sigma USA) as substrates [19]. A slight modification of this method was made in measuring of the liberated ammonia [20]. The activity of xanthine oxidase was measured by the formation of uric acid by using 0.05 mmol/L xanthine (Serva-Germany) as substrate [21]. Tissue proteins were measured by Lowry method [22]. The activity of enzymes was expressed as U/g proteins, and the protein content was expressed as mg/g wet weight (mg/gWW).

### 2.3. Statistical Analysis

 Mean values ± SD are given. Statistical significance was estimated by the Student *t-test*.

## 3. Results

**Figure 1:**
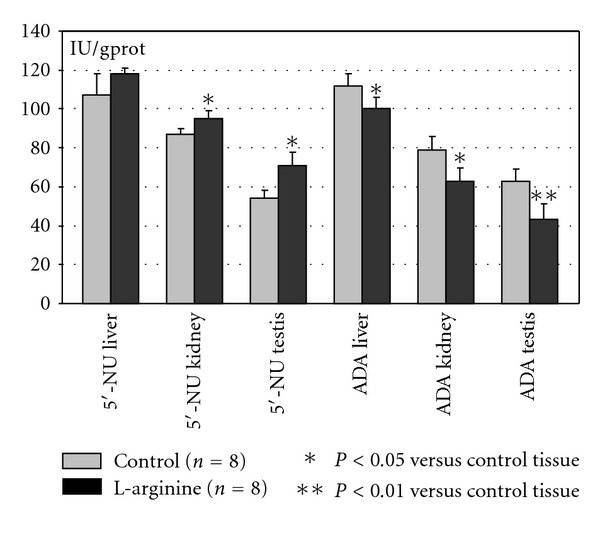
The effect of dietary L-arginine on the activity of 5′-nucleotidase (5′-NU) and adenosine deaminase (ADA) of rat liver, kidney, and testis tissue. The activity of 5′-NU was measured by using 10 mmol/L AMP as substrate, where phosphorus liberation was measured. The activity of ADA was determined by measuring the ammonia liberation by using 4 mmol/L adenosine as substrate. The activity of enzymes was expressed as U/g proteins. Mean values ± SD are given. Each group consisted of 8 animals.

**Figure 2:**
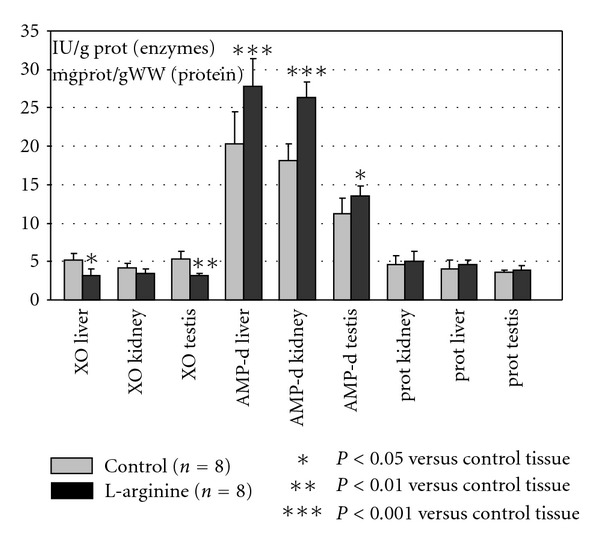
The effect of dietary L-arginine on the activity of AMP deaminase, Xanthine oxidase (XO), and total protein concentration of rat liver, kidney, and testis tissue. The activity of AMP deaminase was determined by measuring the ammonia liberation by using 10 mmol/L AMP as substrate. The activity of xanthine oxidase was measured by the formation of uric acid by using 0.05 mmol/L xanthine as substrate. The activity of enzymes was expressed as U/g proteins. Mean values ± SD are given. Each group consisted of 8 animals.

 The results are shown on the Figures 1 and 2. The activity of 5′-nucleotidase (5′NU) significantly increased in kidney and testis tissue; the activity of ADA significantly decreased in all investigated tissues. Since the first enzyme is involved in adenosine production and former in its degradation, obtained results may point out that the metabolism of adenosine would be directed toward its increased production and limited degradation (Figure 1). The activity of AMP deaminase significantly increased in all tissues, while the activity of XO significantly decreased. It may suggest that the metabolism of adenine nucleotides would be directed to the interconversion process and limited degradation during the L-arginine intake (Figure 2). The mean content of proteins did not change significantly during the L-arginine intake (Figure 2). 

## 4. Discussion

 L-arginine metabolism involves various organs such as the kidney, the muscle, the intestines, the liver, the testis, and the CNS, acting together in an interorgan axis. Dietary supplementation of L-arginine was suggested presumably for three main reasons: its role in the secretion of endogenous growth hormone, its involvement in the synthesis of creatine, and the role in augmenting NO production. NO mediates many of the vasoactive properties of adenosine and may modulate adenosine metabolism. Production of nitric oxide from L-arginine has been implicated in the regulation of steroidogenesis. These aspects of L-arginine supplementation may be discussed in the light of clinical investigations involving antiatherogenic, vasoactive, antioxidant, immunomodulatory effect, and wound-repair activity [13]. L-arginine deficiency could result delay in sexual maturity and development of sterility, impairment of the production of insulin, glucose intolerance, and impaired liver lipid metabolism and detoxification. When administered in high doses, L-arginine stimulates pituitary release of growth hormone and prolactin, pancreatic release of glucagon and insulin, decreased platelet aggregation, and decreased blood pressure. The effect is improved blood circulation in the body and especially in the extremities and in genitalia [8, 10, 17].

 The enzymes of purine metabolism have proved to be particularly sensitive to the effect of dietary L-arginine supplementation (Figures 1 and 2). The activity of 5′-NU significantly increased. Ecto-5′-nucleotidase is mainly located in plasma membrane and its activity is a main source of adenosine production. ADA represents the enzyme involved in recycling of purines and in reducing intracellular accumulation of adenosine [23]. The dynamic ratio of these enzymes (5′-NU/ADA) increased in L-arginine-treated group, suggesting that adenine nucleotide metabolism may be directed toward stimulated adenosine production and increased adenosine pool. Extracellular adenosine may exert several physiological effects by stimulation of specific adenosine receptors via decreasing vascular tone [24–27]. Beside this, the adenosine is an important anti-inflammatory agent [28], which inhibits TNF-*α* production in macrophages and monocytes, suppresses arachidonic acid release and leukotriene biosynthesis in human neutrophils [29], and is shown to act as an endogenous activator of cellular antioxidant enzyme systems [30]. As a regulator of vascular cell proliferation and death, it was a powerful endogenous protector against atherosclerotic and vasoocclusive disorders [12–14, 31–33]. Taking together the multiple actions of both, adenosine or L-arginine, for the regulation of metabolic functions of different organs, it seems that the accumulation of adenosine may reproduce similar effect as that of L-arginine product, NO, and that in some circumstances, they can also act in a synergistic manner [2, 34, 35]. The activity of AMP deaminase, the ATP regenerating enzyme, significantly increased. In relation to the role of AMP deaminase in the interconversion of IMP to ATP or guanine nucleotides and the stabilization of the adenylate energy charge (ATP + 2ADP)/(ATP + ADP + AMP), highly active AMP deaminase may additionally contribute to intracellular ATP regeneration, which depends on the adenylate pool and on the energy charge [12, 14]. Increased activity of AMP deaminase induces the activity of phosphofructokinase and pyruvate kinase, maintaining in this way intermediary metabolism [36].

The L-arginine-induced decrease of XO activity is of particular interest. Xanthine oxidase is a rate-limiting enzyme in terminal step of purine nucleotide degradation. Since the XO represents one of the main sources of free radical production, decreased activity may contribute to the decreased lipid peroxidation by dietary L-arginine supplementation [6].

 In conclusion, L-arginine exerted effect on purine metabolism in liver, kidney, and testis tissue by activation of adenosine production, salvage pathway, and ATP regeneration, which may have the protective effects on male metabolic and reproductive functions.
